# Twin study shows association between monocyte chemoattractant protein-1 and kynurenic acid in cerebrospinal fluid

**DOI:** 10.1007/s00406-019-01042-9

**Published:** 2019-07-13

**Authors:** Viktoria Johansson, Sophie Erhardt, Göran Engberg, Magdalena Kegel, Maria Bhat, Lilly Schwieler, Kaj Blennow, Henrik Zetterberg, Tyrone D. Cannon, Lennart Wetterberg, Christina M. Hultman, Mikael Landén

**Affiliations:** 1 Department of Medical Epidemiology and Biostatistics, Karolinska Institutet, PO Box 281, 171 77 Stockholm, Sweden; 2 Centre for Psychiatry Research, Department of Clinical Neuroscience, Karolinska Institutet and Stockholm Health Care Services, Stockholm County Council, Norra Stationsgatan 69, 11364 Stockholm, Sweden; 3 Department of Physiology and Pharmacology, Karolinska Institutet, Stockholm, Sweden; 4 Protein Diagnostics, Precision Medicine and Genomics, IMED Biotech Unit, AstraZeneca, Gothenburg, Sweden; 5 Department of Psychiatry and Neurochemistry, Institute of Neuroscience and Physiology, Sahlgrenska Academy at the Gothenburg University, Gothenburg, Sweden; 6 Clinical Neurochemistry Laboratory, Sahlgrenska University Hospital, Mölndal, Sweden; 7 Department of Neurodegenerative Disease, Institute of Neurology, University College London, Queen Square, London, UK; 8 UK Dementia Research Institute, London, UK; 9 Department of Psychology, Yale University, New Haven, CT USA; 10 Department of Psychiatry, Yale University School of Medicine, New Haven, CT USA; 11 Department of Clinical Neuroscience, Karolinska Institutet, Stockholm, Sweden; 12 Department of Psychiatry, Icahn School of Medicine at Mount Sinai, New York, NY USA

**Keywords:** Twin study, Cerebrospinal fluid, Biomarker, Kynurenic acid, Monocyte chemoattractant protein-1, Chemokine ligand 2

## Abstract

Preclinical studies indicate a link between the kynurenine pathway and monocyte chemoattractant protein-1 (MCP-1), but there is a lack of clinical studies examining this further. We here perform a secondary analysis of kynurenine metabolites and MCP-1 in cerebrospinal fluid of 23 twins affected from schizophrenia, bipolar disorder or unaffected. We show an association between MCP-1 and kynurenic acid (KYNA), driven by unique environmental influences and a less pronounced association between MCP-1 and tryptophan. No association was detected between MCP-1 and quinolinic acid. Further studies on the mechanism behind the putative relationship between KYNA and MCP-1 are needed.

**Electronic supplementary material:**

The online version of this article (10.1007/s00406-019-01042-9) contains supplementary material, which is available to authorized users.

## Introduction

The kynurenine pathway is the major route for tryptophan (TRP) degradation and gives rise to neuroactive compounds like kynurenic acid (KYNA) and quinolinic acid (QUIN), displaying neuroprotective and neurotoxic properties, respectively [1]. Abnormalities in the kynurenine pathway have been linked to numerous brain disorders [2], including psychiatric disorders such as schizophrenia and bipolar disorder [3].

Monocyte chemoattractant protein 1 (MCP-1), also known as chemokine ligand 2 (CCL2), is involved in the recruitment of macrophages to infection sites within the central nervous system (CNS) [4], and preclinical studies have demonstrated a relationship between MCP-1 and the kynurenine pathway. In mice, for example, reduction of indoleamine 2,3-dioxygenase (IDO)—the mediating enzyme of tryptophan degradation—resulted in lower MCP-1 levels [5, 6], and plasma kynurenine correlated with MCP-1 gene expression within the CNS [7]. In a study on in vitro cells of human monocytes, prior exposure to kynurenine had an enhancing effect on MCP-1-induced transmigration [8]. Although abnormal peripheral levels of MCP-1 have been associated with bipolar disorder [9, 10], and psychosis [9, 11], no studies have explored the relationship between MCP-1 and the kynurenine pathway within the human CNS.

To explore the role of MCP-1 in relation to the kynurenine pathway in a clinical sample, we here conduct a secondary analysis of previously published data. In the original reports, we analyzed CSF levels of TRP, KYNA, QUIN, Interleukin 6 (IL-6), IL-8, and tumor necrosis factor alpha (TNF-α) [12], and MCP-1 [13] in twins with psychiatric morbidity. We found mutual correlations of QUIN, IL-8, and TNF-α, and that higher CSF KYNA correlated with psychotic symptoms and personality traits [12]. Here, we analyze available CSF data from 23 monozygotic (MZ) or dizygotic (DZ) twins affected by schizophrenia or bipolar disorders. First, we estimate correlations between the CSF levels of MCP-1 and KYNA, TRP, QUIN, IL-6, IL-8, and TNF-α. Second, we analyze correlations in the MZ and DZ twin pairs to estimate the genetic and environmental effects.

## Materials and methods

### Study population

From the Schizophrenia and bipolar twin study in Sweden (STAR) [14], we had available kynurenine and MCP-1 CSF data on 25 same-sex individual twins (see flowchart in Supplementary Fig. 1). We excluded two twins due to analysis failure, and for this report, complete data were available from 23 twins (10 complete pairs). The twins were clinically assessed with the Structured Clinical Interviews for DSM-IV Axis I and II (SCID I and II). Information on socioeconomic factors (e.g., education), smoking status, and age at disease onset were available. Zygosity determination (DNA analysis) resulted in 12 MZ and 11 DZ twins [15].

### The sampling of cerebrospinal fluid and blood

The CSF sampling occurred between March 2008 and September 2011 as described previously [16]. Lumbar punctures were performed in the morning by a clinical neurologist. The needle was inserted in vertebral interspace (L3–L5), in a sitting position and 12 mL of CSF was collected, and stored at − 80° Celsius pending analysis. Self-assessment scales for depression [Montgomery–Åsberg Depression Scale (MADRS-S)] and hypomania/mania [Young Ziegler Mania Rating Scale (YMRS)] were administered. Blood samples were drawn at 0800 h with subjects fasting. Height and weight were measured, and body mass index (BMI) calculated.

### Analytical procedures

We refer to the Supplemental information for detailed descriptions of the analytical procedures including CSF/serum albumin ratio, C-reactive protein (CRP) and CSF analyses of MCP-1, KYNA, TRP, QUIN, IL-6, Il-8, and TNF-α.

### Statistical analyses

Sample characteristics are presented as percentages, means (standard deviations), or medians (maximum, minimum-scores). CSF markers were transformed to standardized scores (mean = 0, SD = 1). Associations of mean values between CSF markers were analyzed with linear regression with a cluster-robust sandwich estimator (which accounts for the dependency between the twin pairs when estimating the standard errors). Adjustments were made for sex, age at CSF sampling, psychiatric diagnosis (i.e., schizophrenia with or without affective features, or bipolar disorder), and smoking status. In the twin analyses, for each marker, within-twin pairs differences were calculated (twin 1–twin 2) in complete pairs (*n* = 10). We regressed the within-pair differences for each marker using a conditional linear regression model (fixed effects regression) with a cluster-robust sandwich estimator for the pairs. For multiple testing, we used Bonferroni correction yielding the limit *p* value < 0.003 based on the total amount of CSF markers (*n* = 17) that were previously analyzed. Statistical analyses were performed in STATA 15.1.

### Interpretation

To disentangle shared environmental and genetic mechanisms, associations between the within-pair differences of two markers were analyzed in MZ and DZ pairs separately. An equal or higher regression coefficient in MZ twins than in DZ twins was interpreted as influence from  the unique environment. A higher regression coefficient in DZ twins was interpreted as genetic influences.

### Ethical considerations

The study was approved by the Ethical Review Board, Stockholm (Dnr: 2004-448; 2007-779), and was performed in compliance with the Helsinki Declaration.

## Results

For demographics and clinical characteristics, see Table 1. CSF concentrations of MCP-1, kynurenine metabolites, cytokines, and descriptive statistics for this cohort have been reported previously [12, 13]. The markers were analyzed in a linear regression model in all twins (*n* = 23). After adjustments for age, sex, smoking, and any psychiatric diagnosis (schizophrenia/bipolar disorder), higher MCP-1 was associated with higher KYNA and TRP (Table 2). There was no association between MCP-1 and QUIN. For complete results of all CSF markers, see Supplementary Table 1.

**Table 1:** Demographic and clinical characteristics of the twin sample (*n* = 23 individual twins)

	Monozygotic twins	Dizygotic twins
	*n* = 12^a^	*n* = 11^b^
	*n* (%)	*n* (%)
Sex		
Males	5 (41.67)	8 (72.73)
Females	7 (58.33)	3 (27.27)
Age at sampling, in years, median (min–max)	55 (50–65)	56 (38–58)
Completed education		
Elementary school	4 (33.33)	4 (36.36)
High school (2 years)	3 (25)	1 (9.09)
High school (3 years)	2 (16.67)	2 (18.18)
University	3 (25)	3 (27.27)
Unknown		1 (9.09)
Smoker	4 (33.33)	4 (36.36)
Diagnosis		
Schizophrenia	1 (8.33)	2 (18.18)
Schizophrenia with affective features	2 (16.67)	1 (9.09)
Bipolar disorder	1 (8.33)	1 (9.09)
Not affected	8 (66.67)	7 (63.64)
Type of twin pair		
Concordant schizophrenia	2 (16.67)	2 (18.18)
Discordant schizophrenia	3 (25)	2 (18.18)
Discordant bipolar disorder	3 (25)	3 (27.27)
Not affected	4 (33.33)	4 (36.36)
Age at onset in years, median (min–max)	29.5 (16–55)	28.0 (19–54)
Clinical parameters		
Body mass index, median (min–max)	28.2 (7.5)	29.7 (7.5)
Albumin ratio, mean (SD)	7.3 (3.2)	5.3 (2.1)
CRP, mean (SD)	6.4 (6.7)	4.2 (5.7)
Assessment scales at sampling		
MADRS-S, mean (SD)	2.0 (3.0)	2.7 (3.4)
YMRS, mean (SD)	0.2 (0.4)	1.3 (3.0)
Medication		
Antipsychotics^c^	4 (33.33)	4 (36.36)
Antidepressants	4 (33.33)	4 (36.36)
Antiepileptics	0 (0)	2 (18.18)
Lithium	0 (0)	0 (0)

*SD* standard deviation, *CRP* C-reactive protein, *MADRS-S* self-rated Montgomery–Åsberg Depression Rating Scale, *YMRS* Young Ziegler Mania Rating Scale, *MCP-1* monocyte chemoattractant protein 1, *KYNA* kynurenic acid, *TRP* tryptophan, *QUIN* quinolinic acid, *IL* interleukin, *TNF-α* tumor necrosis factor alpha

^a^Five complete twin pairs. In two twin pairs, CSF data was available from one of the twins in the pair

^b^Five complete twin pairs. In one twin pair, one of the twins participated in the CSF sampling

^c^Types of antipsychotics: haloperidol, levomepromazine, olanzapine, perphenazine, quetiapine, or risperidone

**Table 2:** Association between monocyte chemoattractant protein-1 (MCP-1) and kynurenine metabolites and cytokines in cerebrospinal fluid (CSF)

Biomarkers	*N*	MCP-1	*p* value	MCP-1	*p* value
		Model 1		Model 2	
		Reg. coef. (95% CI)		Reg. coef. (95% CI)	
Tryptophan (TRP)	23	0.40 (0.14, 0.65)	0.006	0.37 (0.16, 0.58)	0.002*
Kynurenic acid (KYNA)	23	0.48 (0.25, 0.70)	0.001*	0.45 (0.19, 0.71)	0.002*
Quinolinic acid (QUIN)	23	0.16 (− 0.15, 0.46)	0.28	0.049 (− 0.29, 0.39)	0.76
Interleukin 6 (IL 6)	23	− 0.22 (− 0.42, − 0.017)	0.036	− 0.087 (− 0.37, 0.20)	0.52
Interleukin 8 (IL-8)	23	0.022 (− 0.32, 0.36)	0.89	− 0.014 (− 0.36, 0.33)	0.93
Tumor necrosis factor alpha (TNF-α)	23	− 0.12 (− 0.58, 0.34)	0.58	− 0.12 (− 0.49, 0.25)	0.50

Results from the linear regression analysis of the mean values in all twins

Model 1: Adjusted for age and sex. Model 2: Adjusted for age, sex, diagnosis of schizophrenia or bipolar disorder and smoking

All variables were standardized [mean = 0, standard deviation (SD) = 1]. Linear regression was applied with a cluster-robust sandwich estimator for the standard errors to account for the twin pair relationships. Results presented as the regression coefficient (Reg. coef.) with 95% confidence intervals (CI)

*Indicates that the *p* values are significant after Bonferroni correction (*p* value < 0.003)

**Fig. 1:**
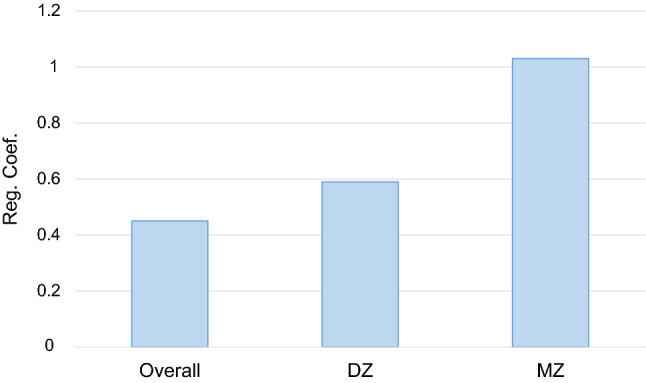
Association between monocyte chemoattractant protein-1 (MCP-1) and kynurenic acid (KYNA) in cerebrospinal fluid (CSF): overall analysis, in dizygotic (DZ) twins and in monozygotic (MZ) twins. The pattern does not indicate a genetic influence of the association between MCP-1 and KYNA. Regression coefficients are presented for regression of the mean values in all twin individuals (overall, *n* = 23), for within pair differences in DZ (dizygotic) twins (*n* = 10) and for within pair differences in MZ (monozygotic) twins (*n* = 10). *p* values: overall: *p* = 0.002, DZ: *p* = 0.002, MZ: *p* = 0.001. All values were standardized before analysis [mean = 0, standard deviation (SD) = 1]

Within-pair differences of MCP-1 and kynurenine metabolites and cytokines were analyzed in the MZ pairs (*n* = 5) taking shared environmental and genetic factors into account, and the DZ pairs (*n* = 5) accounting for shared environmental factors. The within-pair differences between MCP-1 and KYNA were significantly associated, and the regression coefficient was higher in the MZ than in the DZ pairs (Table 3), which remained significant after correcting for multiple testing. In Fig. 1, the regression coefficients for MCP-1 and KYNA are presented from the overall analysis and the DZ and MZ analyses, respectively. Within-pair differences between MCP-1 and TRP were associated in the DZ but not in the MZ pairs (Table 3). Associations found between MCP-1 and TNF-α or IL-8 did not survive adjustments for multiple testing (Table 3). Antidepressant treatment was associated with higher MCP-1 levels (data not shown). To account for this, we excluded twins with ongoing antidepressant medication, which did not change the main results (data not shown).

**Table 3:** Association between monocyte chemoattractant protein-1 (MCP-1) and kynurenine metabolites and cytokines in cerebrospinal fluid (CSF)

CSF-markers	Within-pair differences—MZ pairs (*n* = 5)		Within-pair differences—DZ pairs (*n* = 5)	
	MCP-1		MCP-1	
	Reg. Coef. (95% CI)	*p* value	Reg. Coef. (95% CI)	*p* value
Tryptophan (TRP)	− 0.52 (− 2.32, 1.28)	0.51	0.51 (0.24, 0.79)	0.005
Kynurenic acid (KYNA)	1.03 (0.64, 1.42)	0.001*	0.59 (0.33, 0.85)	0.002*
Quinolinic acid (QUIN)	0.11 (− 0.84, 1.05)	0.79	0.02 (− 3.92, 3.95)	0.99
Interleukin 6 (IL 6)	0.14 (− 0.04, 0.32)	0.12	1.38 (− 4.48, 7.24)	0.57
Interleukin 8 (IL-8)	− 0.49 (− 0.94, − 0.04)	0.037	− 2.06 (− 5.47, 1.34)	0.18
Tumor Necrosis Factor alpha (TNF-α)	− 0.48 (− 0.74, − 0.22)	0.004	− 1.25 (− 3.65, 1.16)	0.24

Results from the conditional linear regression analysis of the differences within the complete twin pairs

All variables were standardized [mean = 0, standard deviation (SD) = 1]. Conditional linear regression was used for analysis, with cluster-robust sandwich estimator for standard errors. Results presented as the regression coefficient (Reg. Coef.) with 95% confidence intervals (CI). We interpreted that a higher coefficient in the MZ pairs than in the DZ pairs, was an effect from unique environmental factors, and a higher coefficient in the DZ pairs than in the MZ pairs as an effect from genetic factors

*Indicates that the *p* values are significant after Bonferroni correction (*p* value < 0.003)

## Discussion

We here report evidence for a link between the inflammatory markers MCP-1 and KYNA in the CNS. We show an overall association between MCP-1 and KYNA in CSF, conceivably driven by unique environmental influences according to the twin analysis. There was a less pronounced association between MCP-1 and TRP overall, but the twin analysis demonstrated that genetic factors may account for the correlation. Notably, we did not find any association between MCP-1 and QUIN.

It has long been known that the immune system activates tryptophan degradation via the kynurenine pathway [17]. Particularly, interferon gamma (IFN-γ), but also neopterin, a marker for IFN-γ activity [18], have been associated with an upregulation of several neuroactive kynurenine metabolites such as KYNA and QUIN in CSF [19, 20]. In accordance with our findings, studies on rodents showed that manipulation of TRP degradation affects MCP-1 levels [7, 5, 6, 8] and MCP-1 seems to play a role in neurotransmission such as dopamine release [21]. Clinical CSF studies on kynurenine metabolites and inflammatory markers are rare. Yet, one study analyzed CSF from hepatitis C-affected patients on interferon alpha (INF-α) treatment, and found trend-level associations between KYNA and MCP-1. Contrasting our findings, they found a correlation between QUIN and MCP-1 [22], but might be due to the massive inflammatory response induced by the INF-α treatment [23].

The major strength of this study is the unique sampling of CSF from twins, which allowed us to disentangle genetic and environmental factors. However, the sample size is small, and some participants were affected by psychiatric conditions and/or had ongoing medication, which may affect the CSF marker levels. The results must, therefore, be considered as indicative and interpreted with caution.

To conclude, by analyzing CSF from twins, we suggest that brain KYNA contributes to higher MCP-1 levels or vice versa. Further studies are required to determine a causal relationship between KYNA and MCP-1.

## Electronic supplementary material

Below is the link to the electronic supplementary material.

Supplementary material 1 (PDF 360 kb)

**Supplementary Fig.** **1**. Flowchart of the included twin sample. CSF = Cerebrospinal fluid. MCP-1 = monocyte chemoattractant protein-1. (PDF 106 kb)

Supplementary material 3 (PDF 208 kb)

## References

[1] Cervenka I, Agudelo LZ, Ruas JL (2017). Kynurenines: tryptophan’s metabolites in exercise, inflammation, and mental health. Science Science.

[2] Chen Y, Guillemin GJ (2009). Kynurenine pathway metabolites in humans: disease and healthy States. Int J Tryptophan Res.

[3] Erhardt S, Schwieler L, Imbeault S, Engberg G (2017). The kynurenine pathway in schizophrenia and bipolar disorder. Neuropharmacology.

[4] Semple BD, Kossmann T, Morganti-Kossmann MC (2010). Role of chemokines in CNS health and pathology: a focus on the CCL2/CCR21 and CXCL8/CXCR21 networks. J Cereb Blood Flow Metab.

[5] Metz R, Smith C, DuHadaway JB, Chandler P, Baban B, Merlo LM, Pigott E, Keough MP, Rust S, Mellor AL, Mandik-Nayak L, Muller AJ, Prendergast GC (2014). IDO2 is critical for IDO1-mediated T-cell regulation and exerts a non-redundant function in inflammation. Int Immunol.

[6] Moheno P, Morrey J, Fuchs D (2010). Effect of dipterinyl calcium pentahydrate on hepatitis B virus replication in transgenic mice. J Transl Med.

[7] Zang X, Zheng X, Hou Y, Hu M, Wang H, Bao X, Zhou F, Wang G, Hao H (2018). Regulation of proinflammatory monocyte activation by the kynurenine-AhR axis underlies immunometabolic control of depressive behavior in mice. Faseb J.

[8] Agudelo LZ, Femenia T, Orhan F, Porsmyr-Palmertz M, Goiny M, Martinez-Redondo V, Correia JC, Izadi M, Bhat M, Schuppe-Koistinen I, Pettersson AT, Ferreira DMS, Krook A, Barres R, Zierath JR, Erhardt S, Lindskog M, Ruas JL (2014). Skeletal muscle PGC-1alpha1 modulates kynurenine metabolism and mediates resilience to stress-induced depression. Cell.

[9] Goldsmith DR, Rapaport MH, Miller BJ (2016). A meta-analysis of blood cytokine network alterations in psychiatric patients: comparisons between schizophrenia, bipolar disorder and depression. Mol Psychiatry.

[10] Jakobsson J, Bjerke M, Sahebi S, Isgren A, Ekman CJ, Sellgren C, Olsson B, Zetterberg H, Blennow K, Palsson E, Landen M (2015). Monocyte and microglial activation in patients with mood-stabilized bipolar disorder. J Psychiatry Neurosci.

[11] Orhan F, Schwieler L, Fatouros-Bergman H, Malmqvist A, Cervenka S, Collste K, Flyckt L, Farde L, Sellgren CM, Piehl F, Engberg G, Erhardt S (2018). Increased number of monocytes and plasma levels of MCP-1 and YKL-40 in first-episode psychosis. Acta Psychiatr Scand.

[12] Kegel ME, Johansson V, Wetterberg L, Bhat M, Schwieler L, Cannon TD, Schuppe-Koistinen I, Engberg G, Landen M, Hultman CM, Erhardt S (2017). Kynurenic acid and psychotic symptoms and personality traits in twins with psychiatric morbidity. Psychiatry Res.

[13] Johansson V, Jakobsson J, Fortgang RG, Zetterberg H, Blennow K, Cannon TD, Hultman CM, Wetterberg L, Landen M (2017). Cerebrospinal fluid microglia and neurodegenerative markers in twins concordant and discordant for psychotic disorders. Eur Arch Psychiatry Clin Neurosci.

[14] Johansson V, Hultman CM, Kizling I, Martinsson L, Borg J, Hedman A, Cannon TD (2019). The schizophrenia and bipolar twin study in Sweden (STAR). Schizophr Res.

[15] Hannelius U, Gherman L, Makela VV, Lindstedt A, Zucchelli M, Lagerberg C, Tybring G, Kere J, Lindgren CM (2007). Large-scale zygosity testing using single nucleotide polymorphisms. Twin Res Hum Genet.

[16] Johansson V, Nybom R, Wetterberg L, Hultman CM, Cannon TD, Johansson AG, Ekman CJ, Landen M (2012). Microscopic particles in two fractions of fresh cerebrospinal fluid in twins with schizophrenia or bipolar disorder and in healthy controls. PLoS One.

[17] Hayaishi O, Yoshida R (1978). Specific induction of pulmonary indoleamine 2,3-dioxygenase by bacterial lipopolysaccharide. Ciba Found Symp.

[18] Widner B, Leblhuber F, Fuchs D (2002). Increased neopterin production and tryptophan degradation in advanced Parkinson’s disease. J Neural Transm (Vienna).

[19] Gostner JM, Geisler S, Stonig M, Mair L, Sperner-Unterweger B, Fuchs D (2019). Tryptophan metabolism and related pathways in psychoneuroimmunology: the impact of nutrition and lifestyle. Neuropsychobiology.

[20] Quist-Paulsen E, Aukrust P, Kran AB, Dunlop O, Ormaasen V, Stiksrud B, Midttun O, Ueland T, Ueland PM, Mollnes TE, Dyrhol-Riise AM (2018). High neopterin and IP-10 levels in cerebrospinal fluid are associated with neurotoxic tryptophan metabolites in acute central nervous system infections. J Neuroinflammation.

[21] Guyon A, Skrzydelski D, De Giry I, Rovere C, Conductier G, Trocello JM, Dauge V, Kitabgi P, Rostene W, Nahon JL, Melik Parsadaniantz S (2009). Long term exposure to the chemokine CCL2 activates the nigrostriatal dopamine system: a novel mechanism for the control of dopamine release. Neuroscience.

[22] Raison CL, Dantzer R, Kelley KW, Lawson MA, Woolwine BJ, Vogt G, Spivey JR, Saito K, Miller AH (2010). CSF concentrations of brain tryptophan and kynurenines during immune stimulation with IFN-alpha: relationship to CNS immune responses and depression. Mol Psychiatry.

[23] Raison CL, Borisov AS, Majer M, Drake DF, Pagnoni G, Woolwine BJ, Vogt GJ, Massung B, Miller AH (2009). Activation of central nervous system inflammatory pathways by interferon-alpha: relationship to monoamines and depression. Biol Psychiatry.

