# Supplementary material for: Twin study shows association between monocyte chemoattractant protein-1 and kynurenic acid in cerebrospinal fluid
Source: Eur Arch Psychiatry Clin Neurosci. 2019 Jul 13;270(7):933–8. doi: 10.1007/s00406-019-01042-9 (PMC7474706; doi:10.1007/s00406-019-01042-9)
Supplement: Supplementary file 1 — Supplementary material 1 (PDF 360 kb) [file 406_2019_1042_MOESM1_ESM.pdf]

Twin study shows association between MCP-1 and KYNA in cerebrospinal fluid

Supplementary information on laboratory analyses

Analysis of MCP-1

CSF MCP-1 concentration was measured using the MSD® Human MCP-1 Ultra-Sensitive Kit, as described by the manufacturer (Meso Scale Discovery, Gaithersburg, MD, USA). The analyses were performed in one round of analyses using one batch of reagents by board-certified laboratory technicians who were blinded to clinical information.

Analysis of kynurenic acid

The analysis of CSF KYNA was performed using an isocratic reversed-phase high-performance liquid chromatography (HPLC) system, including a dual-piston, high-liquid delivery pump (Bischoff, Leonberg, Germany), a ReproSil-Pur C18 column (4 × 150 mm, Dr. Maisch GmbH, Ammerbuch, Germany), and a fluorescence detector (Jasco FP-2020, Hachioji City, 154 Japan.) with an excitation wavelength of 344 nm and an emission wavelength of 398 nm (18 nm bandwidth). A mobile phase of 50 mM sodium acetate (pH 6.2, adjusted with acetic acid) and 7.0% acetonitrile was pumped through the reversed-phase column at a flow rate of 0.5 mL/min. Samples of 50 µL were manually injected into a Rheodyne injector (Rohnert Park, CA). Zinc acetate (0.5 M not pH adjusted) was delivered after the column by a peristaltic pump (P-500; Pharmacia, Uppsala, Sweden) at a flow rate of 0.10 mL/min. Signals from the fluorescence detector were transferred to a computer for analysis with Datalys Azur (version 4.6.0.0; http://datalys.net). The retention time of KYNA was about 7–8 minutes. Initially, the sensitivity of the system was verified by analysis of a standard mixture of KYNA with concentrations from 0.5 to 30 nM, which resulted in a linear standard plot. To verify the reliability of this method, some samples were analyzed in duplicate, and the mean intraindividual variation was below 5%.

Analysis of tryptophan and quinolinic acid

The analysis of QUIN and TRP in CSF was performed using liquid chromatography tandem mass spectrometry and labelled internal standards ($^{13}C_3{}^{15}N_1$-QUIN; Synfine research Inc., Ontario, Canada and D_5-TRP; Sigma-Aldrich, St. Louis, MO, USA), Waters Acquity HPLC system and a Xevo TQ-S triple quadrupole mass spectrometer. The mass spectrometer operated in positive ionization MS/MS mode using mass spectral transition for QUIN and TRP at m/z 168 > 106; 205 > 118 and for the internal standard 172 > 110; 210 > 123. Further details on the method can be found in [4]. Quality control samples was included to verify the reliability of this method.

Analysis of cytokines

CSF samples for cytokine analysis had previously been thawed twice. IL-1β, IL-6, IL-8, and TNF-α were quantified in CSF using a customized Human Ultra-Sensitive 4-Plex Kit (MesoScale Discovery®, Gaithersburg, MD, USA) in 2012. The assays were analyzed as per the manufacturers protocol (http://www.mesoscale.com), with the modification of a longer primary incubation time (overnight at 4°C) and a sample volume of 50 μL. Intra-assay coefficient of variation was below 20% for all analytes presented. The limits of detection (LOD) in our analysis were: IL-1β (0.19 pg/mL), IL-6 (0.05 pg/mL), IL-8 (0.04 pg/mL), and TNF-α (0.08 pg/mL). IL-1β was found to be below LOD in all samples analyzed and therefore not included in the statistical analysis.

Analysis of blood–CSF barrier function and C-reactive protein

Albumin levels in CSF and serum were measured by immunonephelometry on a Beckman Image Immunochemistry system (Beckman Instruments, BeckmanCoulter, Brea, CA, USA). The Swedish Board for Accreditation and Conformity Assessment (SWEDAC) accredited the method. Experienced and board-certified laboratory technicians who were blinded to clinical information performed all measurements. Intra- and interassay coefficients of variation were below 10%. To assess the blood-CSF barrier function, the ratio between albumin concentration in CSF (mg/L) and serum (g/L) was calculated.

Levels of C-reactive protein (CRP) was analyzed in blood using an immunoturbimetric assay (Siemens Healthcare Diagnostics Inc. and Beckman Coulter Inc.) with a quantification limit of 0.2 mg/L.

Laboratories

The Clinical Neurochemistry Laboratory in Mölndal, Sweden performed analyses of the CSF concentrations of MCP-1, as well as albumin levels in CSF and serum. Analyses of CSF KYNA and cytokines were performed at the Department of Physiology and Pharmacology, Karolinska Institutet, Stockholm, Sweden. Analyses of CSF QUIN and TRP were performed at the Department of Translational Science Center, AstraZeneca, Science for Life Laboratory, Stockholm, Sweden. C-reactive protein (CRP) was analyzed at Unilabs, Stockholm, Sweden.
