# Supplementary figures and images for: Twin study shows association between monocyte chemoattractant protein-1 and kynurenic acid in cerebrospinal fluid
Source: Eur Arch Psychiatry Clin Neurosci. 2019 Jul 13;270(7):933–8. doi: 10.1007/s00406-019-01042-9 (PMC7474706; doi:10.1007/s00406-019-01042-9)

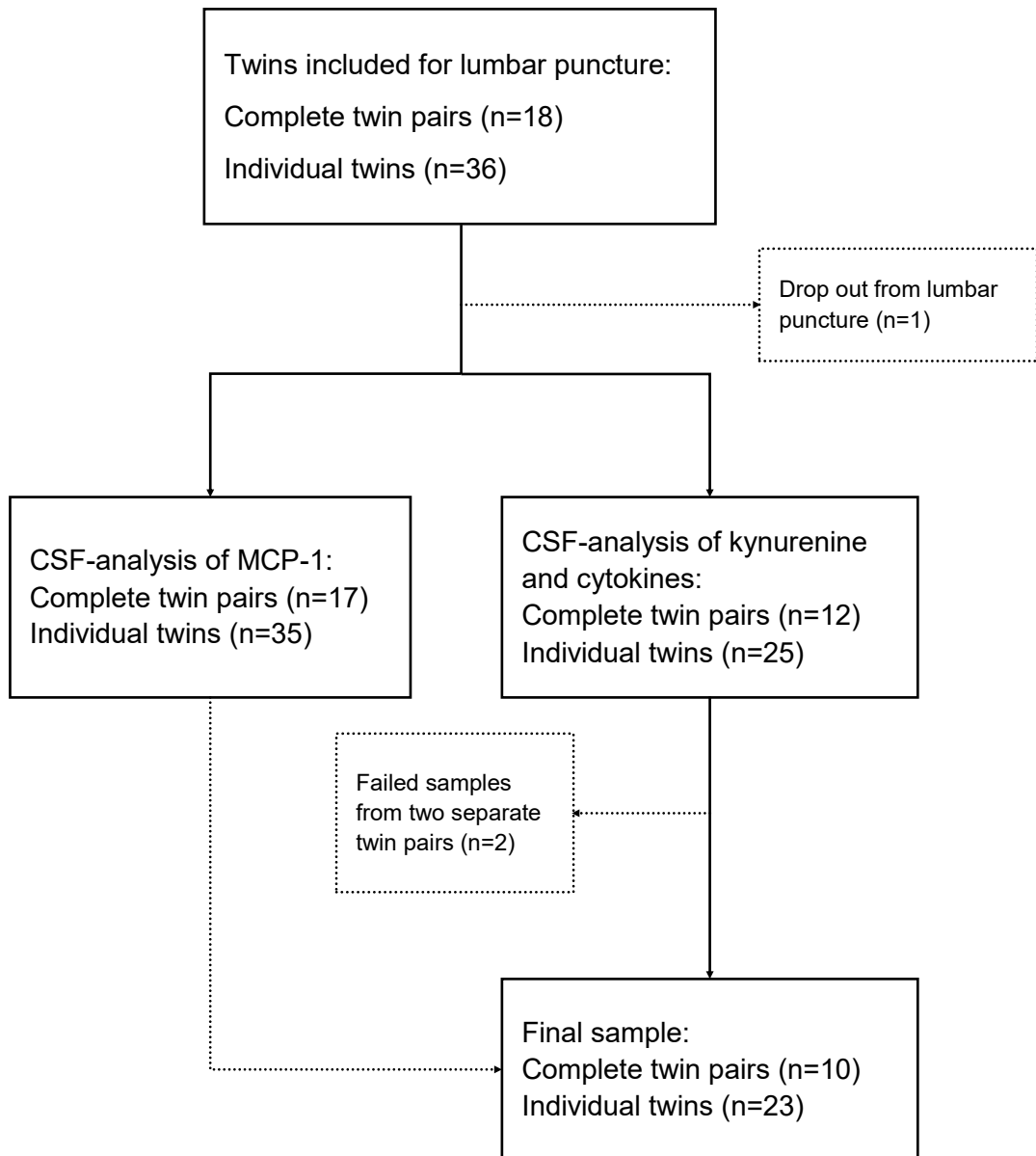

Supplement: Supplementary file 2 — Supplementary Fig. 1. Flowchart of the included twin sample. CSF = Cerebrospinal fluid. MCP-1 = monocyte chemoattractant protein-1. (PDF 106 kb) [file 406_2019_1042_MOESM2_ESM.pdf]
