# Supplementary material for: Twin study shows association between monocyte chemoattractant protein-1 and kynurenic acid in cerebrospinal fluid
Source: Eur Arch Psychiatry Clin Neurosci. 2019 Jul 13;270(7):933–8. doi: 10.1007/s00406-019-01042-9 (PMC7474706; doi:10.1007/s00406-019-01042-9)
Supplement: Supplementary file 3 — Supplementary material 3 (PDF 208 kb) [file 406_2019_1042_MOESM3_ESM.pdf]

Supplementary table 1. Associations between all cerebrospinal fluid (CSF) markers included in this report in all twins (n=23). Results from the linear regression analysis of the mean values in all twins.

	**KYNA**	**QUIN**	**TRP**	**IL-6**	**IL-8**	**TNF-α**
**MCP-1**	**Reg. coef. 0.48, p=0.001**	Reg. coef. 0.16, p=0.283	**Reg. coef. 0.40, p=0.006**	**Reg. coef. -0.22, p=0.036**	Reg. coef. 0.022, p=0.888	Reg. coef. -0.12, p=0.575
**KYNA**	***	Reg. coef. 0.15, p=0.496	**Reg. coef. 0.79, p=0.001**	Reg. coef. -0.097, p=0.369	Reg. coef. -0.21, p=0.355	Reg. coef. 0.21, p=0.304
**QUIN**		***	Reg. coef. -0.16, p=0.417	Reg. coef. -0.0011, p=0.992	**Reg. coef. 0.86, p=0.0001**	**Reg. coef. 0.81, p=0.0001**
**TRP**			***	Reg. coef. -0.14, p=0.118	Reg. coef. -0.32, p=0.182	Reg. coef. -0.31, p=0.147
**IL-6**				***	Reg. coef. 0.19, p=0.241	Reg. coef. 0.25,, p=0.268
**IL-8**					***	**Reg. coef. 0.97 p=0.0001**
**TNF-α**						***

Note: MCP-1 = Monocyte chemoattractant protein 1, KYNA = Kynurenic acid, QUIN = Quinolinic acid, TRP = Tryptophan, IL-6 = Interleukin 6, IL-8 = Interleukin 8, and TNF-α = Tumor necrosis factor-alpha.

Linear regression with a cluster-robust sandwich estimator for the standard errors to account for the twin pair relationships. Results presented as the regression coefficient (Reg. coef.) and p-values. Adjusted for age and sex.

Parts of the results were published elsewhere (Kegel, M.E., et al., Kynurenic acid and psychotic symptoms and personality traits in twins with psychiatric morbidity. Psychiatry Res, 2017. **247**: p. 105-112).
